# Core symbionts, age at inoculation and diet affect colonization of the bumblebee gut by a common bacterial pathogen

**DOI:** 10.1111/1365-2656.70029

**Published:** 2025-04-03

**Authors:** Annika S. Nelson, McKenna J. Larson, Tobin J. Hammer

**Affiliations:** ^1^ Department of Biology Texas Christian University Fort Worth Texas USA; ^2^ Department of Ecology and Evolutionary Biology University of California Irvine California USA

**Keywords:** *Bombus impatiens*, colonization resistance, microbiome, microbiota, *Serratia marcescens*

## Abstract

1. Microbes shape the health of bumblebees, an important group of pollinators, including species of conservation concern. Most microbial research on bumblebees has focused on eukaryotic and viral pathogens or the core gut microbiome, a community of host‐specialized bacterial symbionts that helps protect hosts against eukaryotic pathogens.
2. Bumblebees also harbour a third class of microbes: non‐core gut bacteria, which are non‐host specific and vary among individuals. Understanding their functional role and how they interact with core symbionts is important for bumblebee ecology and management.
3. We surveyed non‐core bacteria in wild bumblebee workers (*Bombus impatiens*) and conducted laboratory experiments with gnotobiotic *B. impatiens* to examine factors shaping colonization by a focal non‐core bacterium (*Serratia marcescens*) and its consequences for bee health.
4. Non‐core bacteria, including *Serratia*, frequently occur at high abundance in wild bumblebees, with roughly half of individuals harbouring at least 10% non‐core gut bacteria. Experiments showed that *Serratia marcescens* better colonizes the gut when bees are inoculated early (within 1 day of adult emergence) and the core gut microbiome is disrupted. A mixed wildflower pollen diet facilitated the highest level of infection compared with two monofloral pollen treatments. We also provide evidence that *Serratia* is pathogenic: exposing bees with disrupted gut microbiomes to *Serratia* strongly reduced lifespan and, as a result, also reduced total reproduction.
5. These results have three important implications: first, non‐core bacteria are widespread in wild bumblebees, and some species are opportunistic pathogens. Second, the core gut microbiome plays a crucial role in protecting against these pathogens. Third, the timing of inoculation relative to bee age, as well as diet, is a key factor controlling bacterial pathogen colonization of the gut. Overall, these findings suggest that gut bacterial health could be an important target for monitoring and managing bumblebee health.

## Graphical abstract

This paper examines ecological interactions within the bumblebee gut microbiome and between the microbiome and host using field sampling and controlled experiments. We demonstrate that non‐core bacteria occur frequently in high abundance in wild bumblebee gut microbiomes. One common non‐core bacterium, *Serratia*, reduces bee lifespan and reproduction. The core microbiome, bee age and pollen diet can protect against *Serratia* infection.

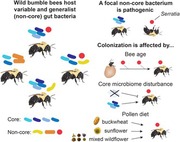

## 1 INTRODUCTION

Bumblebees (*Bombus* spp.) are key pollinators of wild plants and crops (Drummond, 2012; Strange, 2015), yet many populations are declining rapidly (Cameron et al., 2011). Several factors have been implicated, of which disease plays a major role (Cameron et al., 2016; Meeus et al., 2011). Bumblebees are also pivotal models for understanding the evolutionary ecology of microbiomes and disease (Koch & Schmid‐Hempel, 2011b, 2012; Schmid‐Hempel, 2001). Thus, understanding the identities and ecologies of bumblebee‐associated microbes has implications for both conservation and fundamental research on host–microbe interactions.

Most research on bumblebee‐associated microbes has focused on two groups – eukaryotic or viral pathogens, and core gut bacterial symbionts. Pathogens like *Crithidia, Vairimorpha, Apicystis* and deformed wing virus are widespread in bumblebees and can reduce fitness (Cordes et al., 2012; Figueroa, Sadd, et al., 2023; Fürst et al., 2014; Genersch et al., 2006; Pascall et al., 2021). Since the foundational work of Koch and Schmid‐Hempel (2011a, 2011b, 2012) demonstrated that bumblebees host a specialized set of gut bacteria that help defend against *Crithidia* infection, research has expanded to include the core gut microbiome and its interactions with eukaryotic pathogens. Core bacterial symbionts, which colonize the gut of adult bees, include *Snodgrassella, Gilliamella, Schmidhempelia* and bumblebee‐specific lineages of *Lactobacillus* and Bifidobacteriaceae (Hammer, Le, Martin, et al., 2021). They are remarkably conserved across populations and species and are distinctive from those associated with other insects (Hammer, Le, Martin, et al., 2021; Koch & Schmid‐Hempel, 2011a; Kwong, Medina, et al., 2017; Villabona et al., 2023). Currently, their main known ecological function is in reducing infection by the pathogen *Crithidia bombi* (Koch & Schmid‐Hempel, 2011b, 2012; Mockler et al., 2018).

A third, less‐studied group of bumblebee‐associated microbes is non‐core gut bacteria, which are found across multiple *Bombus* species and continents (Cariveau et al., 2014; Li et al., 2015; Meeus et al., 2015; Villabona et al., 2023). Non‐core bacteria occur in many host‐associated microbiomes and are defined as being non‐host‐specific and highly variable in abundance and composition among individuals (Neu et al., 2021; Perlman et al., 2022; Shade & Handelsman, 2012). While core bacteria usually dominate bumblebee gut microbiomes, some individuals are colonized almost exclusively by non‐core bacteria (Li et al., 2015; Villabona et al., 2023), likely acquired from the environment. However, the ecology and function of non‐core bacteria remain poorly understood. Some taxa could be opportunistic pathogens, although this possibility has been largely overlooked due to the nearly exclusive focus on eukaryotic pathogens and viruses (Figueroa, Sadd, et al., 2023; Macfarlane et al., 1995). Alternatively, non‐core bacteria could have minimal effects or even benefit the host (Cariveau et al., 2014; Mockler et al., 2018; Praet et al., 2018). Better understanding the ecology and function of non‐core bacteria has the potential to inform bumblebee management and conservation. For example, some species could be developed into probiotics for managed bumblebees (Motta et al., 2022; Peixoto et al., 2022). For wild bees, non‐core bacteria might indicate population health. Overall, knowing the ecological factors regulating non‐core bacteria could inform bee management.

Most knowledge about non‐core gut bacteria in bees comes from the Western honeybee (*Apis mellifera*). Honeybees share many core bacterial symbionts with bumblebees (Kwong & Moran, 2016) and host a variety of non‐core bacteria, including some opportunistic pathogens (Fünfhaus et al., 2018; Lang et al., 2022). For example, *Serratia marcescens* strains isolated from honeybee guts can cause sepsis and death when ingested (Braglia et al., 2024; Raymann et al., 2018). The core gut microbiome helps protect against these pathogens (Lang et al., 2022; Steele et al., 2021). However, it remains unclear how this information may apply to bumblebees, given important differences between the two groups in their biology and microbiomes (Hammer, Le, Martin, et al., 2021).

The factors influencing non‐core bacterial colonization of bumblebees are poorly understood. Two key, interconnected factors are endogenous immunity and the core gut microbiome. Gut epithelial immunity is known to regulate microbial colonization in bumblebees and other insects (Deshwal & Mallon, 2014; Engel & Moran, 2013). The core gut microbiome could also provide colonization resistance (i.e. inhibit infection; Caballero‐Flores et al., 2023) against non‐core bacteria, as seen with *Crithidia bombi* (Koch & Schmid‐Hempel, 2011b, 2012; Mockler et al., 2018). In bumblebees, immune gene expression and core gut bacterial abundance both increase within the first 4 days of adulthood (Hammer et al., 2023). Therefore, bee age at inoculation may influence non‐core bacterial colonization through both independent and interactive effects of host immunity and the core gut microbiome.

Diet is another factor that could affect gut colonization by non‐core bacteria. As generalists, bumblebees forage from different plants over space and time (Goulson, 2003). Plant species vary in the nutritional and chemical composition of their nectar and pollen (Palmer‐Young et al., 2019; Vaudo et al., 2015, 2016). Variation in floral resources and foraging preferences could affect non‐core bacteria colonization. For example, sunflower pollen has been shown to reduce infection by *Crithidia bombi* in *Bombus impatiens* (Figueroa, Sadd, et al., 2023; Fowler et al., 2019, 2022; Giacomini et al., 2018). Pollen composition can also have more subtle effects on bumblebee gut bacterial diversity (Fowler et al., 2024). Research on honeybees supports similar interactions between pollen composition and the gut microbiome, potentially mediated by immunity (Castelli et al., 2020; Motta et al., 2023; Palmer‐Young et al., 2017).

In this study, we examined the distribution of non‐core bacteria in wild bumblebees, factors shaping gut colonization by a focal non‐core bacterium and consequences for bee health. We conducted field surveys and laboratory experiments to ask: (1) How prevalent are non‐core bacteria in wild bumblebee (*Bombus impatiens*) workers? (2) Do the core microbiome, age at inoculation and diet influence colonization of gnotobiotic *B. impatiens* workers by a common non‐core bacterium (*Serratia*)? and (3) What are the effects of *Serratia* on *B. impatiens* survival and reproduction? Our findings highlight non‐core bacteria as an underrecognized factor influencing bumblebee populations and suggest possible applications for conservation and management.

## 2 MATERIALS AND METHODS

### 2.1 Study organisms

The Common Eastern BumbleBee (*Bombus impatiens*) is one of the most widespread bumblebees in eastern North America (Novotny et al., 2021) and a key pollinator of many wild and cultivated plants (Strange, 2015). *Bombus impatiens* is frequently reared in captivity and is currently the only commercially available bumblebee species in North America, making it a useful study system for integrating laboratory experiments with field observations. *Serratia* is a common environmental bacterium found in bumblebee guts, often linked to depleted core gut microbiomes or toxin exposure (Li et al., 2015; Rothman et al., 2020). *Serratia* is an opportunistic pathogen of many insects, including honeybees (Burritt et al., 2016; Fünfhaus et al., 2018; Raymann et al., 2018). Here, we used *Serratia* as a focal non‐core gut bacterium. Experiments used a labelled strain of *S. marcescens* KZ11, which was originally isolated from a honeybee gut (*Apis mellifera*) (Raymann et al., 2018) and genetically modified to carry a kanamycin resistance marker (Steele et al., 2021).

### 2.2 Non‐core bacteria prevalence in wild bumblebees

To assess the prevalence of non‐core bacteria in wild *B. impatiens* workers, we sampled bees from 20 field sites near Madison, WI, Glencoe, IL and Amherst, MA, in June–August 2023. Permits were not required to collect from these sites. We caught 91 *B. impatiens* foragers with nets and aseptically dissected their gut (hindgut and midgut). Each gut was homogenized with a sterile pestle and stored in 20% glycerol in phosphate‐buffered saline (PBS). Samples were shipped overnight on ice to the University of California, Irvine, where they were stored at −70°C. We extracted DNA and performed 16S rRNA gene amplicon sequencing (detailed below) to measure non‐core bacteria prevalence, defined as all taxa outside of the core genera *Snodgrassella, Gilliamella, Candidatus Schmidhempelia, Bifidobacterium, Bombiscardovia* and *Lactobacillus* (Hammer, Le, Martin, et al., 2021; Kwong & Moran, 2015).

### 2.3 Core microbiome, inoculation timing and diet effects on *Serratia* colonization

**FIGURE 1:**
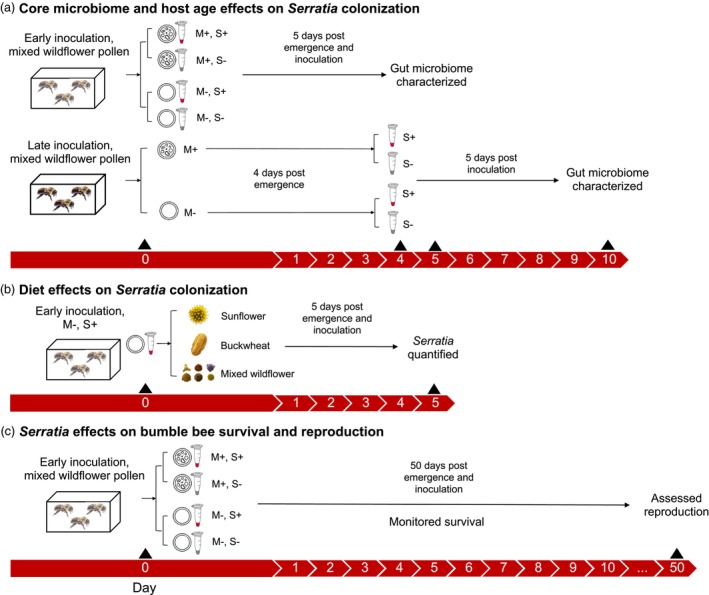
Design of experiments testing for (a) core gut microbiome and inoculation timing effects on colonization by a non‐core bacterium (*Serratia*), (b) pollen diet effects on *Serratia* colonization and (c) *Serratia* effects on bumblebee survivorship and reproduction. The timelines indicate the number of days after adults eclosed from surface‐sterilized pupal cocoons. Bees were placed in core microbiome transplant (‘M+’) or depletion (‘M−’) treatments, *Serratia* (‘S+’) or *Serratia*‐free control (‘S−’) treatments in which bees were inoculated early (Day 0) or late (Day 4), and three different diet treatments (sunflower, buckwheat or mixed wildflower pollen).

To examine whether the core gut microbiome and timing of inoculation influence non‐core bacteria colonization of *B. impatiens*, we conducted laboratory experiments with gnotobiotic bees and the labelled strain of *Serratia marcescens* (experimental setup shown in Figure 1a). These laboratory experiments did not require ethical approval. We established age‐controlled gnotobiotic microcolonies of *B. impatiens* workers sourced from three commercial colonies (Beneficial Insectary, Biobest Group, CA, USA). Because larvae shed their gut lining during metamorphosis, newly emerged adults have few gut bacteria prior to interacting with nestmates and hive substrates (Hammer & Moran, 2019; Zhang & Zheng, 2022). Thus, to create gnotobiotic bees, we removed clumps of pupal cocoons from each colony and surface‐sterilized them in 0.18% bleach for 90 s (Hammer, Le, & Moran, 2021; Näpflin & Schmid‐Hempel, 2018). Pupae were kept under sterile conditions at 35°C and monitored daily. Newly emerged adults (<1 day old and nearly free of gut symbionts) were transferred in groups of three to microcolonies (Klinger et al., 2019) consisting of sterilized 16 oz. plastic containers with bedding. Each microcolony was maintained at 27°C and provided filter‐sterilized 50% sucrose syrup and sterile pollen dough (ground and ethylene oxide‐sterilized honeybee‐collected pollen from wildflowers of unknown composition (Koppert Biological Systems, MI, USA) mixed with sterile 50% sucrose syrup) ad libitum.

To test whether the core gut microbiome influences *Serratia* colonization, we applied core microbiome transplant (‘M+’) and depletion (‘M−’) treatments to microcolonies when they were established (Figure 1a). We inoculated M+ bees with gut homogenate from non‐sterile bees from the same original colony (Koch & Schmid‐Hempel, 2012; Mockler et al., 2018). Indoor‐reared bees generally only host core symbionts (Hammer et al., 2023; Meeus et al., 2015), and 16S rRNA gene sequencing confirmed that M+ bees were dominated by core symbionts (see Section 3; Figure S1.4). To prepare the gut homogenate, six workers from the source colony were anaesthetized on ice before removing, pooling and homogenizing their guts in 500 μL PBS. M+ microcolonies were inoculated by pipetting 20 μL of this homogenate onto the sterile pollen dough, which the bees could freely consume (Mockler et al., 2018; Powell et al., 2023). M− microcolonies received 20 μL sterile PBS as a control. M− bees were not completely microbe‐free; some core and non‐core gut bacteria were detectable from sequencing, perhaps due to incomplete sterilization of pupal clumps, food or housing (Figure S1.4). However, total bacterial abundance (approximated using the total number of 16S rRNA gene sequencing reads) was much lower for M− compared with M+ bees (Figure S1.3).

We exposed microcolonies to *Serratia* when bees were either <1 day old (‘early’ treatment; 12 M+ and 10 M− microcolonies) or 4–5 days old (‘late’ treatment; 10 M+ and 9 M− microcolonies; Figure 1a), as this period shows strong shifts in the core gut microbiome and immune gene expression (Hammer et al., 2023). For the *Serratia* treatment (‘S+’), we pipetted approximately 10^8^ colony‐forming units (CFUs) of an overnight *S. marcescens* culture in 20 μL PBS onto the pollen dough. As a control (‘S−’), four early M+, five early M−, five late M+ and five late M− microcolonies received 20 μL sterile PBS solution. Several S− bees ended up being colonized by the labelled *Serratia* strain, suggesting occasional cross‐contamination between cages. Furthermore, 16S rRNA gene sequences belonging to *Serratia* were evident in some S− bees; these likely originated from our labelled strain but could also represent contamination by other *Serratia* strains in the rearing environment. However, across all experiments, *Serratia* occurred at significantly higher frequency and abundance in S+ bees (see Section 3).

Five days after *Serratia* treatment, we assessed colonization by anaesthetizing each bee on ice and removing the gut (Figure 1a). Each gut was homogenized with a sterile pestle in 1 mL PBS, serially diluted and plated on LB agar with 20 μg/mL kanamycin, with four technical replicates per sample. After 24 h incubation at 30°C, we counted CFUs to estimate *S. marcescens* abundance per gut (detection range of 2.5 × 10^2^ to 6 × 10^7^ CFUs). The remaining gut homogenate was stored at −20°C for later DNA extraction and 16S rRNA gene amplicon sequencing (detailed below) to further assess colonization by core bacteria and *Serratia*.

In a separate experiment, we tested how diet influences *Serratia* colonization (Figure 1b). We established microcolonies of gnotobiotic microbiome‐depleted (M−) *B. impatiens* workers sourced from two commercial colonies, as described above. Each microcolony received filter‐sterilized 50% sucrose syrup ad libitum and sterile pollen dough in one of three treatments: monofloral buckwheat (*Fagopyrum esculentum*; nine microcolonies), monofloral sunflower (*Helianthus annuus*; nine microcolonies) or the polyfloral wildflower pollen described above (seven microcolonies; Figure 1b; LoCascio et al., 2019). Buckwheat and sunflower pollen were sourced from Henan Zhuoyu Bee Products/Bee Farm Biotechnology Co., Ltd., Henan, China, and from Bee Farm Biotechnology Co., Ltd., Henan, China, respectively, and were sterilized by gamma irradiation (8 kGy). Sterility of all pollen types was confirmed by plating on LB and Columbia blood agar. On the day when microcolonies were established, they were all treated with *Serratia* (i.e. all in the ‘early’ inoculation treatment; Figure 1b). Five days later, we assessed *Serratia* colonization as described above and estimated recent pollen consumption as the number of pollen grains per gut (Figure 1b). To do so, we pipetted 10 μL gut homogenate into a Neubauer improved haemocytometer and counted all pollen grains in two separate technical replicates under 100X magnification, similar to Fowler et al. (2020). The number of pollen grains reflects both recent pollen consumption and defecation.

### 2.4 *Serratia* effects on bumblebee survival and reproduction

To test the effects of *Serratia* on *B. impatiens* health, we measured the survival and reproduction of adult workers in a 2 × 2 factorial design manipulating the core microbiome (M+/M−) and *Serratia* (S+/S−), as described above (Figure 1c). Bees were sourced from one commercial colony, and treatments were imposed on the day microcolonies were established (‘early’ inoculation treatment; Figure 1c). Microcolonies received sterile 50% sucrose solution and pollen dough ad libitum and were monitored every 48–72 h for survival and adult male production, as dominant bumblebee workers separated from the colony often lay haploid (male) eggs (Klinger et al., 2019). All dead workers and new adult males were immediately removed from microcolonies. Reproductive output was measured at the end of the experiment (46–50 days, depending on the microcolony; Figure 1c) by counting the total number of eggs, larvae, pupae and adult males that were produced by each microcolony.

### 2.5 Library preparation, sequencing and 16S rRNA amplicon data processing

We extracted genomic DNA (gDNA) from gut homogenates of the 91 field‐collected workers and 173 workers from the *Serratia* colonization experiment. First, 250 μL of each gut homogenate was placed in a tube containing ~0.5 mL of 0.1‐mm silica zirconia beads and 750 μL ZymoBIOMICS Lysis Solution. Samples were processed in a bead beater at 1800 rpm for 2 min, left to rest at room temperature for 1 min, and then bead‐beaten again for 2 min (Powell et al., 2014). Lysed samples were stored at −70°C until DNA extraction, which was performed using the ZymoBIOMICS 96 DNA Kit. Two extraction blanks and two technical replicates of the ZymoBIOMICS microbial community standard were also included. Extracted gDNA was PCR‐amplified using barcoded universal primers targeting the V4 region (515F/806R) of the 16S rRNA gene (Caporaso et al., 2023). Amplification was performed with 23 μL 1X Platinum Hot Start PCR Master Mix, 1 μL barcoded primer and 1 μL gDNA. The thermal profile consisted of initial denaturation at 94°C for 3 min, followed by 35 cycles of denaturation at 94°C for 45 s, annealing at 50°C for 60s and elongation at 72°C for 90s, and terminated by a final elongation at 72°C for 10 min. PCR products were cleaned and normalized using SequalPrep normalization plates. Libraries (including two PCR no‐template controls) were pooled and sequenced on an Illumina MiSeq (2 × 400 bp reads) at the UC Irvine Genomics Research and Technology Hub. Raw reads are available in the European Nucleotide Archive (PRJEB79856).

Sequences were processed using QIIME 2 v2023.9 (Bolyen et al., 2019). DADA2 was used for denoising, truncating reads to 240 bases, filtering chimeric sequences and constructing the amplicon sequence variant (ASV) table (Callahan et al., 2016). Sequencing resulted in a total of 7.3 million reads, 787 ASVs and a mean of 24,906 reads per sample. Taxonomy was assigned to ASVs using the q2‐feature‐classifier (Bokulich et al., 2018) classify‐sklearn using a naïve Bayes classifier trained with the SILVA v138 reference database (Bokulich et al., 2018; Yilmaz et al., 2014). Some ASVs were unclassified or identified only to the Domain level. In these cases, we manually classified the most abundant ASVs (top 100, >99% of all reads) using BLAST (Altschul et al., 1990). For ASVs with 100% match to multiple taxa, we assigned a putative identification as the genus with the greatest number of matches.

To identify potential contaminants, we used the ‘isContaminant()’ function in the ‘decontam’ package (Davis et al., 2018) in R v4.1.1 (R Core Team, 2024). The ‘prevalence’ method identified contaminants based on their prevalence in blanks and PCR no‐template controls compared with true samples. We identified 44 contaminants, which were removed from the ASV table, along with any ASVs with fewer than 100 total reads. We confirmed that all eight expected bacterial taxa were present in both technical replicates of the microbial community standard and comprised >95% of reads. The final ASV table, taxonomy and metadata are available from the Dryad Data Repository: https://doi.org/10.5061/dryad.qbzkh18t1 (Nelson et al., 2025).

### 2.6 Statistical analysis

To examine non‐core bacteria prevalence in wild bumblebee microbiomes, we constructed a linear model (‘LM’) with the proportion of non‐core reads per sample (square root‐transformed) as the response variable and sampling site/period as the fixed effect. We visualized microbiome composition across sampling site/period using non‐metric multidimensional scaling (‘NMDS’) with the Bray–Curtis dissimilarity index. We tested for statistically significant differences in composition using a permutational analysis of variance (‘PERMANOVA’) with 999 permutations.

To determine whether core microbiome and inoculation timing influence *Serratia* colonization, we constructed two linear mixed effects models (‘LMER’). The first model used the number of *S. marcescens* CFUs per gut (*ln* + 1 transformed) as the response, while the second model used the proportion of 16S rRNA gene sequences that were *Serratia* per gut as the response. Both models included the fixed effects of *Serratia* treatment, core microbiome treatment, inoculation timing treatment and colony ID, along with the interaction between core microbiome and inoculation timing. Microcolony ID was a random effect.

To test whether diet affects *Serratia* colonization, we constructed a LMER with *S. marcescens* CFUs per gut (*ln* + 1 transformed) as the response variable, pollen diet treatment and colony ID as fixed effects, and microcolony ID as a random effect. We also investigated whether differences in colonization could have been driven by pollen consumption rates using a separate LMER. The number of pollen grains per gut (*ln* transformed) was the response, and pollen treatment and colony ID were fixed effects, while microcolony ID was a random effect.

We examined how *Serratia* affects *B. impatiens* survival and reproduction with multiple models. For survival, we constructed a Cox proportional hazards model with Firth's penalized likelihood. Bee survival was the response, with core microbiome treatment, *Serratia* treatment and their interaction as fixed effects. For reproduction, we constructed an LMER with total reproduction in each microcolony as the response. Core microbiome treatment, *Serratia* treatment, and their interaction were fixed effects, and microcolony establishment date was a random effect. To test whether the total reproductive output of a microcolony could have simply been driven by differences in bee survival, we constructed an additional LMER that also included as a covariate the summed number of days all bees were alive in a microcolony.

Statistical analyses were conducted in R v4.1.1 (R Core Team, 2024). LMs and LMERs were constructed using the ‘lm()’ function in the ‘stats’ package (R Core Team, 2024) and the ‘lmer()’ function in the ‘lme4’ package (Bates et al., 2015). To assess the significance of fixed effects, we conducted *F* tests (for LMs) or Wald chi‐squared tests with Type III sums of squares (for LMERs) using the ‘Anova()’ function in the ‘car’ package (Fox & Weisberg, 2019). The NMDS ordination and PERMANOVA were conducted using the ‘metaMDS()’ and ‘adonis2()’ functions (respectively) in the ‘vegan’ package (Oksanen et al., 2020). For the Cox proportional hazards model, we used the ‘coxphf()’ function in the ‘coxphf’ package (Heinze et al., 2020) and obtained chi‐square values to test for the significance of effects using the ‘summary()’ function (R Core Team, 2024). Nonsignificant interaction terms were removed from models before testing for the significance of fixed effects. For significant main effects with more than two levels, we conducted Tukey tests using the ‘glht()’ function in the ‘multcomp’ package (Hothorn et al., 2008). Effect sizes were calculated from estimated marginal means using the ‘emmeans()’ function in the ‘emmeans’ package (Lenth, 2021).

## 3 RESULTS

### 3.1 Non‐core bacteria prevalence in wild bumblebees

**FIGURE 2:**
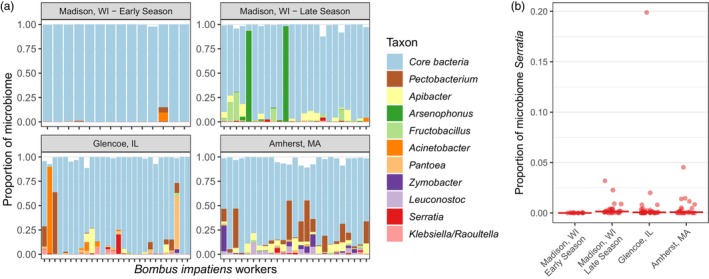
Non‐core bacteria, including *Serratia*, are common in *Bombus impatiens*. (a) Gut microbiome composition (16S rRNA gene sequence data), with each bar corresponding to a bee collected from one of four sampling sites/periods. Core taxa (*Candidatus Schmidhempelia, Gilliamella, Snodgrassella, Lactobacillus, Bifidobacterium* and *Bombiscardovia*) are shown as a single colour to better visualize the ten most common non‐core genera (but are shown individually in Figure S1.2). White space corresponds to rare non‐core taxa not included in the legend. (b) *Serratia* relative abundance is shown on the *y*‐axis for each site (*x*‐axis), with each point corresponding to an individual bee. Here, data from panel A are replotted to better visualize the focal non‐core taxon used in experiments. Solid horizontal lines are medians.

Across the wild *B. impatiens* workers, 43 individuals (47.3%) harboured at least 10% non‐core bacteria in their gut microbiome (Figure 2a). *Serratia*, the non‐core bacterium that was the focus of laboratory experiments, occurred in 67% of wild bees, though usually at low relative abundance (Figure 2b). The proportion of non‐core bacteria per gut differed across sites/sampling periods (*F*_1,3_ = 10.07, *p* < 0.001; Figure 2a). In early summer in Madison, WI, worker gut microbiomes consisted of 0.6 ± 0.9% (mean ± 1SE) non‐core bacteria, which was significantly lower than the proportion in late summer in Madison (12.4 ± 3.1%; *t* = 3.81, *p* = 0.001) or mid‐summer in Glencoe, IL (10.4 ± 2.6%; *t* = 3.50, *p* = 0.004) or Amherst, MA (21.9 ± 4.0%; *t* = 5.47, *p* < 0.001; Figure 2a). Relative abundances of non‐core bacteria did not differ among late summer Madison or mid‐summer Glencoe or Amherst bees (*t* ≤ 2.48, *p ≥* 0.069 in all tests). Additionally, gut microbiome composition significantly differed across sampling sites/sampling periods (*F*_3,87_ = 10.1, *p* < 0.001; Figure S1.1). Relative abundances of eight of the 10 most abundant non‐core genera (*Pectobacterium*, *Apibacter*, *Arsenophonus, Fructobacillus, Pantoea, Zymobacter, Leuconostoc* and *Klebsiella/Raoultella*) significantly differed across sites/sampling periods (Figure S1.2).

### 3.2 Core microbiome, inoculation timing and diet effects on *Serratia* colonization

**FIGURE 3:**
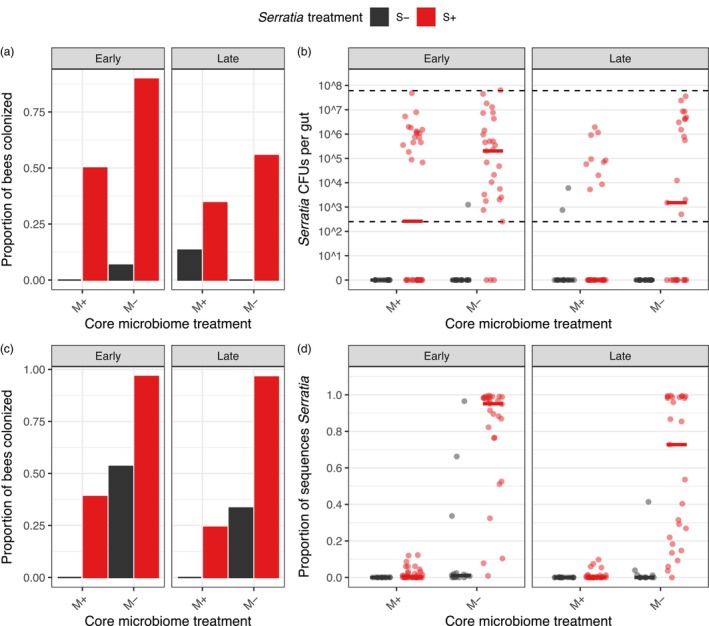
Core microbiome and timing of inoculation influence *Serratia* colonization rates. (a) Proportion of bees with detectable *Serratia* colony‐forming units (CFUs), (b) number of *Serratia* CFUs, (c) proportion of bees with >1% 16S rRNA gene sequences composed of *Serratia*, and (d) proportion of 16S rRNA gene sequences composed of *Serratia*. Note that panels (a and c) are binary detection data, and panels (b and d) are numeric values of the same data. Inoculation timing (‘early’ vs. ‘late’) is shown in separate panels, core microbiome treatment is on the x‐axis (‘M+’ for core microbiome transplant and ‘M−’ for microbiome depletion) and *Serratia* treatment is represented by colour (‘S+’ for bees with *Serratia* and ‘S−’ for the *Serratia*‐free control). Solid horizontal lines are medians. Dashed horizontal lines are the limits of CFU detection.

In the experiment testing for core microbiome and inoculation timing effects on colonization, *Serratia* treatment increased the number of *Serratia* CFUs per gut by >4000‐fold (*χ* ^2^ = 82.94, *p* < 0.001; Figure 3a), demonstrating the effectiveness of inoculation. *Serratia* was 93% less abundant in the core microbiome transplant treatment (*χ* ^2^ = 6.41, *p* = 0.011) but 13‐fold more abundant in bees treated early (versus late; χ ^2^ = 6.33, *p* = 0.012; Figure 3a). Source colony also affected colonization (*χ* ^2^ = 47.40, *p* < 0.001). However, the interaction between microbiome treatment and inoculation timing was not significant (*χ* ^2^ = 0.19, *p* = 0.666; Figure 3a).

16S rRNA gene sequences yielded similar but not identical results, as these data are proportional (rather than absolute) abundances (Figure 3b; Figure S1.3). The proportion of *Serratia* sequences per gut was 30‐fold greater in the *Serratia* treatment (*χ* ^2^ = 23.80, *p* < 0.001; Figure 3b). The core microbiome transplant reduced *Serratia* relative abundance by 113% (*χ* ^2^ = 69.03, *p* < 0.001), although the timing of inoculation had no detectable effect (*χ* ^2^ = 1.38, *p* = 0.240; Figure 3b). There was also no detectable effect of source colony (*χ* ^2^ = 2.88, *p* = 0.237) or an interaction between core microbiome and inoculation timing (*χ* ^2^ = 2.28, *p* = 0.131; Figure 3b).

**FIGURE 4:**
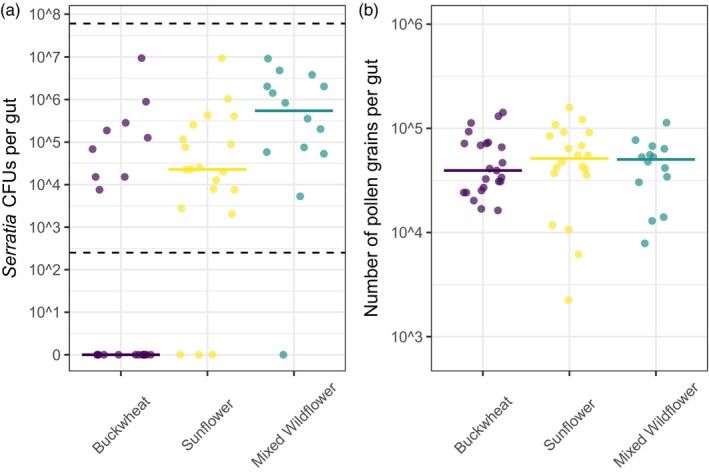
Pollen diet treatment influences *Serratia* colonization but not pollen consumption. (a) Number of *Serratia* colony‐forming units (CFUs; *y*‐axis) and (b) number of pollen grains per gut (*y*‐axis) in three pollen diet treatments (*x*‐axis). Solid horizontal lines are medians. Dashed horizontal lines are the limits of CFU detection.

In the experiment testing for effects of diet on colonization, there were significant effects of pollen diet treatment (*χ* ^2^ = 10.68, *p* = 0.005) and source colony (*χ* ^2^ = 4.11, *p* = 0.043) on the number of *Serratia* CFUs per gut (Figure 4a). *Serratia* colonization was >2000‐fold higher in bees fed wildflower compared with buckwheat pollen (*Z* = 3.20, *p* = 0.004). It was also on average 66‐fold higher in bees fed sunflower versus buckwheat (*Z* = 2.02, *p* = 0.107) and 30‐fold higher in bees fed wildflower versus sunflower pollen (*Z* = 2.37, *p* = 0.317), although these differences were not statistically significant. There was no detectable effect of pollen treatment (*χ* ^2^ = 0.28, *p* = 0.868) or source colony (*χ* ^2^ = 0.03, *p* = 0.873) on the number of pollen grains per gut (Figure 4b).

### 3.3 *Serratia* effects on bee survival and reproduction

**FIGURE 5:**
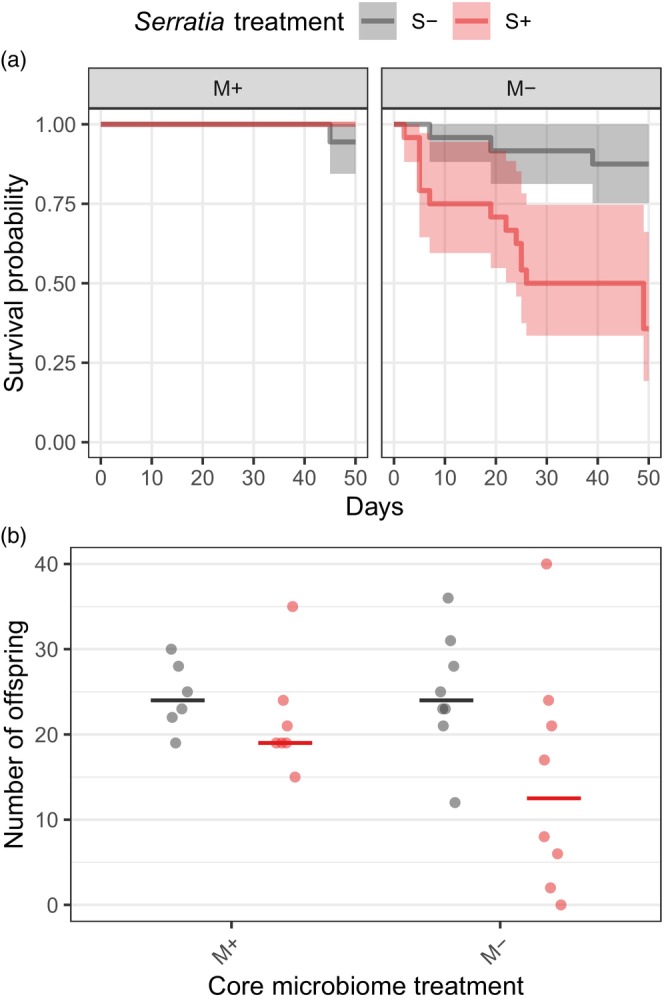
*Serratia* reduces *B. impatiens* survivorship and, as a result, reproduction. (a) Probability of bee survival (*y*‐axis) and (b) total number of offspring produced by microcolonies (*y*‐axis) in core microbiome transplant (‘M+’) or depletion (‘M−’) treatments (panels in a and *x*‐axis in b) and with *Serratia* inoculation (‘S+’) or PBS control treatments (‘S−’; colour). Solid horizontal lines are medians.

*Serratia* reduced *B. impatiens* survival and, as a consequence, reproduction. *Serratia* significantly reduced bee survival (*χ* ^2^ = 10.84, *p* < 0.001), and there was a nonsignificant trend for such effects to be stronger in the absence of the core gut microbiome (*χ* ^2^ = 3.53, *p* = 0.060; Figure 5a), although there was no detectable main effect of core microbiome treatment (*χ* ^2^ = 0.41, *p* = 0.503; Figure 5a). Furthermore, *Serratia* reduced total reproductive output of microcolonies by 28% (*χ* ^2^ = 4.29, *p* = 0.038). Reproduction did not depend on core microbiome treatment (*χ* ^2^ = 1.08, *p* = 0.299) or the interaction between *Serratia* and core microbiome treatment (*χ* ^2^ = 1.23, *p* = 0.268; Figure 5b). After accounting for the significant effect of the number of days each bee was alive (summed across all bees in the microcolony) (*χ* ^2^ = 9.88, *p* = 0.002), the effect of *Serratia* on reproduction was no longer significant (*χ* ^2^ = 0.53, *p* = 0.467). This suggests that *Serratia* does not reduce reproductive rates of individual bees directly but instead reduces total reproductive output of microcolonies through reductions in worker lifespan.

## 4 DISCUSSION

Using field surveys and laboratory experiments, we investigated the factors shaping how non‐core bacteria, a poorly understood class of gut symbionts, colonize and affect the health of bumblebees. Microbial ecology research often focuses on the core microbiome—the set of taxa consistently present among individuals or populations—because of its presumed functional importance (Neu et al., 2021). Social bees, including bumblebees, have a clearly definable core gut microbiome comprising a few lineages of host‐specialized bacteria (Hammer, Le, Martin, et al., 2021; Kwong, Medina, et al., 2017; Kwong & Moran, 2016). This study focuses on the non‐core bacteria, those that are inconsistently present among bumblebees and are not bumblebee‐specialized, instead being characteristic of flowers, other insects or diverse environmental substrates (e.g. Czajkowski et al., 2011; Nováková et al., 2009; Russell & McFrederick, 2022). We note that the concept of core versus non‐core microbes may not easily apply to other hosts, including solitary bees (Voulgari‐Kokota et al., 2019), that have inherently variable and environmentally acquired microbiomes.

We found that non‐core bacteria frequently occur at high abundance in individual wild bumblebees, which is concordant with prior work on *B. impatiens* (Cariveau et al., 2014). We also found that the relative abundance of common non‐core taxa was highly variable across sites and sampling periods. This variability could be driven by multiple factors, such as differences in individual bee or colony age (Hammer et al., 2023; Parmentier et al., 2016) or bacterial abundance in flowers (von Arx et al., 2019). In extreme cases, a subset of individuals (both in this and other studies) has disrupted gut microbiomes, with almost exclusively non‐core taxa (Li et al., 2015; Villabona et al., 2023). This pattern appears to be widespread across bumblebee species and sampling locations, demonstrating a need to understand the role of non‐core gut bacteria in bumblebee health.

Laboratory experiments demonstrated that the core gut microbiome provides colonization resistance against a focal non‐core gut bacterium (*Serratia*). Inoculation with core symbionts reduced both the absolute (assessed from CFU counts) and relative abundance (assessed from 16S rRNA gene sequences) of *Serratia*. Previously, the core gut microbiome's primary known function in bumblebees was in reducing infection by the eukaryotic pathogen *Crithidia* (Koch & Schmid‐Hempel, 2011b, 2012; Mockler et al., 2018). We show that protection from *Serratia* colonization, a core microbiome function documented in honeybees (Lang et al., 2022; Steele et al., 2021), is conserved in bumblebees. Thus, stressors that disrupt the core microbiome (e.g. agrochemicals; Motta et al., 2018) may render bumblebees vulnerable to opportunistic pathogen infection. While the mechanism of protection is unclear, core gut symbionts of honeybees are known to modify the physicochemical environment within the gut (Zheng et al., 2017) and upregulate immune gene expression (Kwong, Mancenido, et al., 2017). Some core bumblebee gut bacteria form a biofilm on the hindgut wall (Hammer, Le, Martin, et al., 2021), creating a physical barrier that may also inhibit colonization.

We found that bees are less susceptible to *Serratia* colonization when exposed later after adult emergence, suggesting that the colonization window for non‐core bacteria narrows as hosts mature. This could in part be explained by the succession of the core gut microbiome during early adulthood (Hammer et al., 2023). However, there was no detectable interaction between core gut microbiome treatment and inoculation timing in this study, suggesting that shifts in host resistance to *Serratia* colonization occur independently from changes in the core gut microbiome. As workers age, they may develop stronger endogenous immune and barrier defences in the gut (Hammer et al., 2023), possibly contributing to elevated colonization resistance.

We found that pollen diet influences *Serratia* colonization, although in an unexpected way. Colonization was highest with polyfloral, intermediate with monofloral sunflower and lowest with monofloral buckwheat pollen. In contrast, sunflower pollen dramatically reduces infection of *B. impatiens* by the eukaryotic pathogen *Crithidia bombi* relative to bees fed buckwheat or mixed wildflower pollen (Figueroa, Fowler, et al., 2023; Fowler et al., 2022). This effect is driven by spines on sunflower pollen exines, which inhibit *C. bombi* attachment to bee hindguts (Figueroa, Sadd, et al., 2023), potentially by speeding up gut transit (Giacomini et al., 2022). Our findings suggest that this inhibitory effect does not extend to bacteria. Pollen nutritional or chemical composition may instead play a more important role. Sunflower and buckwheat pollen are both low in protein content (Nicolson & Human, 2013; Yang et al., 2013), and poor nutrition can impair bumblebee immunity (Roger et al., 2017). Although a monofloral diet with low protein content could be expected to reduce immune defence and increase pathogen colonization (Brunner et al., 2014; Castelli et al., 2020), we found the opposite pattern. *Serratia* growth may depend on host nutritional status, which we expect to be lower on the monofloral diets. Prior work on bumblebees and honeybees has shown that limiting pollen nutrients reduces infection by eukaryotic pathogens (Conroy et al., 2016; Logan et al., 2005). Alternatively, secondary metabolites in buckwheat pollen (e.g. phenolics; Nešović et al., 2020) might directly inhibit *Serratia*. While the mechanism is unclear, our findings demonstrate that diet plays a role in facilitating or inhibiting non‐core bacterial colonization in bumblebees.

Our study also shows that non‐core bacteria can act as opportunistic bumblebee pathogens. *Serratia* reduced bumblebee survival and, as a result, also reduced the total reproductive output of microcolonies, aligning with previous findings that *Serratia* is a generalist pathogen (Burritt et al., 2016; Raymann et al., 2018). Other non‐core bacteria in wild bumblebees, including *Pectobacterium, Arsenophonus, Klebsiella* and *Leuconostoc*, can have parasitic or pathogenic effects on insects (Basset et al., 2000; Hiebert et al., 2020; Insua et al., 2013; Wilkes et al., 2012), suggesting that they may play a similar role in bumblebees. This is significant, as research on bacterial pathogens has been identified as an urgent priority for bumblebee management and conservation (Figueroa, Sadd, et al., 2023). Because non‐core gut bacteria are so common, they co‐occur with other biotic and abiotic stressors (e.g. eukaryotic pathogens, pesticides, lack of floral resources), with the potential for compounding effects (Braglia et al., 2024). We must therefore deepen our understanding of the ecology of non‐core gut bacteria and their effects on host health. While not all non‐core bacteria are likely pathogenic, there is a need to more broadly assess how non‐core bacteria affect bumblebee health, at both the individual and colony levels.

The *Serratia* strain used in our experiments was originally isolated from a honeybee (Raymann et al., 2018; Steele et al., 2021). The fact that it readily colonizes bumblebees in the laboratory suggests that this pathogen could be transmitted between bee species and perhaps between managed and wild populations. Interspecific pathogen transmission presents a major challenge to bee conservation, but nearly all research on bee pathogen spillover has focused on eukaryotic parasites and viruses (Dalmon et al., 2021; Murray et al., 2013; Otterstatter & Thomson, 2008). This study demonstrates that we must also consider the spillover of bacterial pathogens.

In summary, we have shown that non‐core bumblebee gut bacteria include opportunistic pathogens whose colonization success depends on the timing of inoculation, the core gut microbiome and diet. This has significant conservation and agricultural implications, as monitoring and targeting non‐core bacterial pathogens of bumblebees may be important for management. Future work could evaluate whether non‐core bacterial prevalence is a useful indicator of wild bumblebee population health. Effects of pesticides or other stressors on the core gut microbiome and bacterial pathogens should be evaluated. Promoting the core gut microbiome, through probiotics or planting specific wildflowers, could be a strategy to enhance bee resistance to pathogens. Finally, by showing that the core bumblebee gut microbiome provides resistance to opportunistic bacterial pathogens, this work also contributes to our understanding of gut microbiome ecology in social bees.

## AUTHOR CONTRIBUTIONS

Annika S. Nelson and Tobin J. Hammer conceived the ideas and designed the methodology. Annika S. Nelson and McKenna J. Larson collected the data. Annika S. Nelson analysed the data. Annika S. Nelson led the writing of the manuscript. All authors contributed critically to the drafts and gave final approval for publication.

## CONFLICT OF INTEREST STATEMENT

The authors have no conflicts of interest.

## STATEMENT OF INCLUSION

All authors were engaged early on with the research and study design to ensure that the diverse sets of perspectives they represent were considered from the onset. Fieldwork was conducted in consultation with researchers local to the regions where we worked.

## Supporting information


**Figure S1.1** The community composition of gut bacteria in field‐collected *Bombus impatiens* workers varies across sites/sampling periods.
**Figure S1.2.** Non‐core bacteria, including *Serratia*, are common in wild bumblebee (*Bombus impatiens*) workers.
**Figure S1.3.** Core microbiome and *Serratia* treatments influence the total number of reads.
**Figure S1.4.** Core microbiome and inoculation timing influence *Serratia* colonization rates.

## Data Availability

Raw sequence data have been uploaded to the European Nucleotide Archive (PRJEB79856). All other data and R scripts are available from the Dryad Digital Repository: https://doi.org/10.5061/dryad.qbzkh18t1 (Nelson et al., 2025).
