# Supplementary material for: Core symbionts, age at inoculation and diet affect colonization of the bumblebee gut by a common bacterial pathogen
Source: J Anim Ecol. 2025 Apr 3;94(5):985–98. doi: 10.1111/1365-2656.70029 (PMC12056351; doi:10.1111/1365-2656.70029)
Supplement: Supplementary file 1 — Figure S1.1 The community composition of gut bacteria in field‐collected Bombus impatiens workers varies across sites/sampling periods. Figure S1.2. Non‐core bacteria, including Serratia, are common in wild bumblebee (Bombus impatiens) workers. Figure S1.3. Core microbiome and Serratia treatments influence the total number of reads. Figure S1.4. Core microbiome and inoculation timing influence Serratia colonization rates. [file JANE-94-985-s001.docx]

**Appendix S1.**

**Figure S1.1.** The community composition of gut bacteria in field-collected *Bombus impatiens* workers varies across sites/sampling periods. Bray-Curtis dissimilarities among samples are visualized as a non-metric multidimensional scaling (NMDS) plot. Ellipses represent 95% confidence intervals for group centroids ($\pm$ 1 SE). Final stress = 0.16.


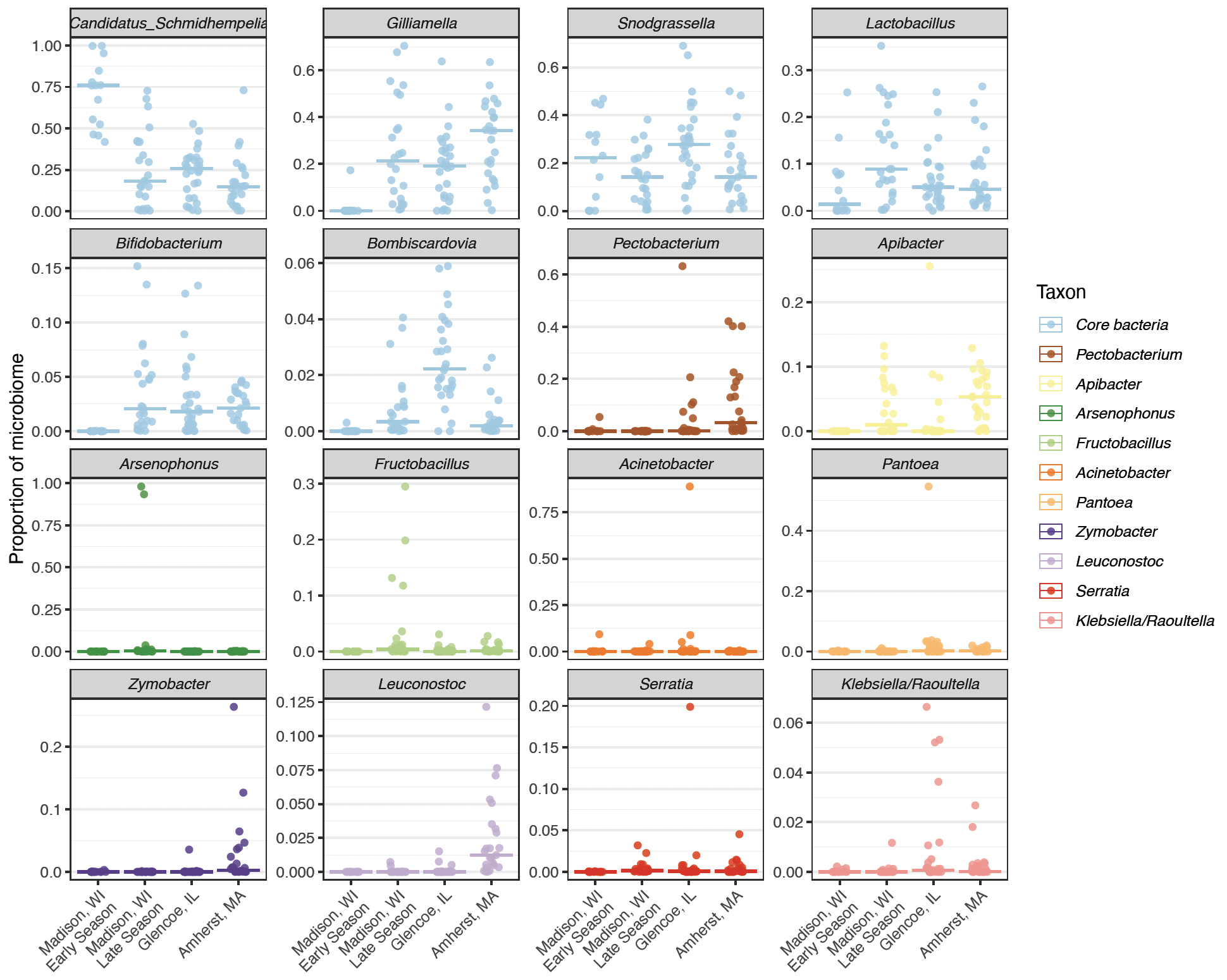


**Figure S1.2.** Non-core bacteria, including *Serratia*, are common in wild bumble bee (*Bombus impatiens*) workers. The proportion of gut microbiome 16S rRNA gene sequences (y-axis) comprising the most abundant core and non-core bacterial genera in each site/sampling period (x-axis). Medians are shown as solid horizontal lines.

**
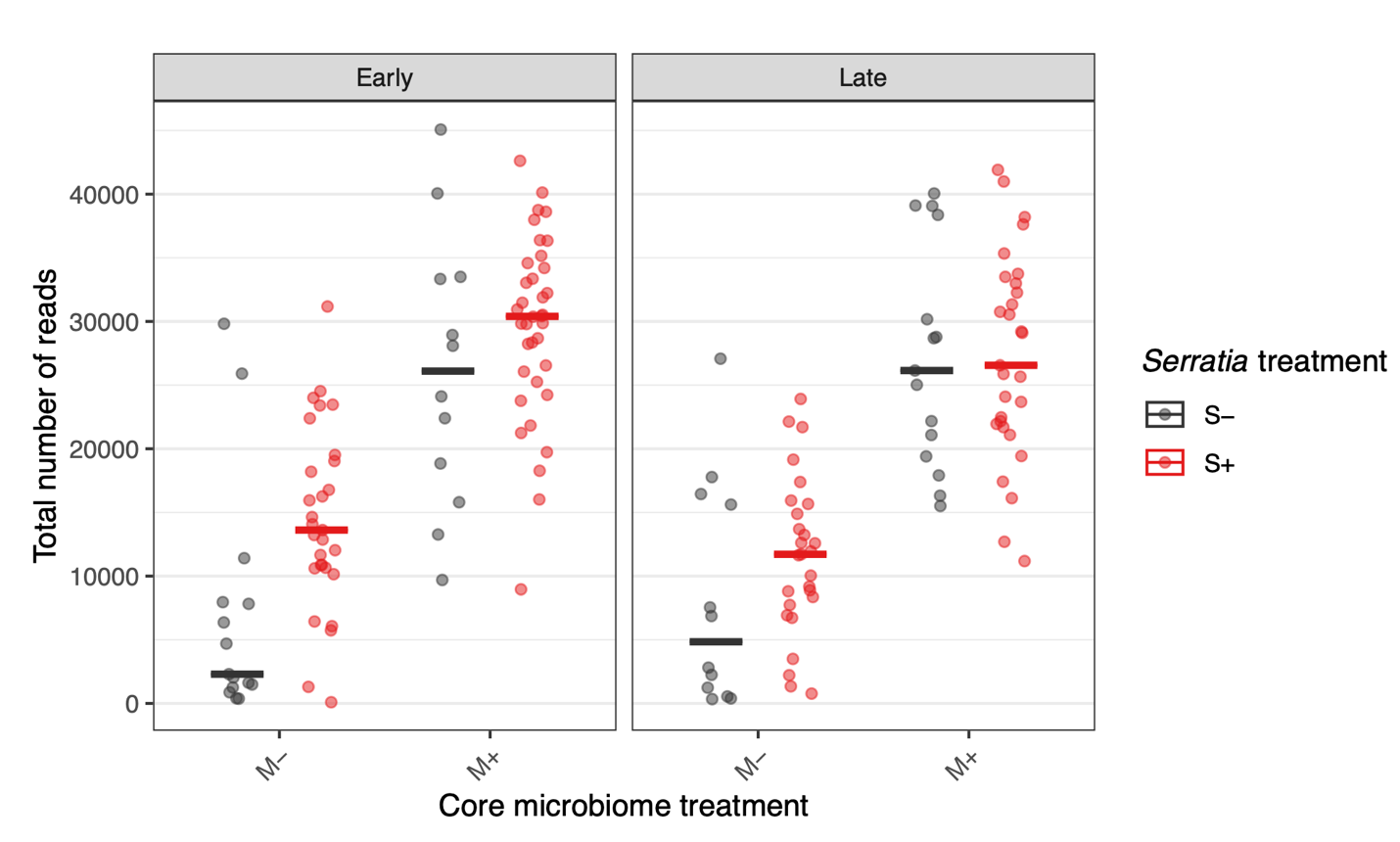
**

**Figure S1.3.** Core microbiome and *Serratia* treatments influence the total number of reads. The total number of reads (from 16S rRNA gene sequence data) is shown on the y-axis, with each point corresponding to an individual bee. Core microbiome treatment is shown on the x-axis (“M+” for core microbiome transplant and “M-” for microbiome depletion), *Serratia* treatment is indicated by color (“S+” for bees with *Serratia* and “S-” for the *Serratia*-free control), and timing of inoculation is shown in separate panels (“Early” versus “Late”). Solid horizontal lines are medians.

**Figure S1.4.** Core microbiome and inoculation timing influence *Serratia* colonization rates. Gut microbiome composition (16S rRNA gene sequence data) is shown, with each bar corresponding to an experimental bee. Panels depict bees in different core microbiome, inoculation timing, and *Serratia* treatments. M+/- refers to bees that received a core microbiome transplant versus sterile buffer. S+/- refers to bees that received an inoculation of *Serratia* versus sterile buffer. Core taxa (*Candidatus Schmidhempelia*, *Snodgrassella, Lactobacillus, Gilliamella, Bifidobacterium,* and *Bombiscardovia*) are shown as various shades of blue.
